# Mechanism of Hepatitis B Virus cccDNA Formation

**DOI:** 10.3390/v13081463

**Published:** 2021-07-27

**Authors:** Lei Wei, Alexander Ploss

**Affiliations:** 110 Lewis Thomas Laboratory, Department of Molecular Biology, Princeton University, Washington Road, Princeton, NJ 08544, USA; leiw@princeton.edu

**Keywords:** hepatitis B virus, HBV, viral replication, cccDNA biogenesis, rcDNA, DNA repair

## Abstract

Hepatitis B virus (HBV) remains a major medical problem affecting at least 257 million chronically infected patients who are at risk of developing serious, frequently fatal liver diseases. HBV is a small, partially double-stranded DNA virus that goes through an intricate replication cycle in its native cellular environment: human hepatocytes. A critical step in the viral life-cycle is the conversion of relaxed circular DNA (rcDNA) into covalently closed circular DNA (cccDNA), the latter being the major template for HBV gene transcription. For this conversion, HBV relies on multiple host factors, as enzymes capable of catalyzing the relevant reactions are not encoded in the viral genome. Combinations of genetic and biochemical approaches have produced findings that provide a more holistic picture of the complex mechanism of HBV cccDNA formation. Here, we review some of these studies that have helped to provide a comprehensive picture of rcDNA to cccDNA conversion. Mechanistic insights into this critical step for HBV persistence hold the key for devising new therapies that will lead not only to viral suppression but to a cure.

## 1. Overview of HBV Life Cycle and cccDNA Biogenesis

The hepatotropic HBV belongs to the *Hepadnaviridae* family and is a blood-borne pathogen. Over a third of the world’s population has been exposed to HBV, leading to 257 million chronic infections and 887,000 deaths per year due to decompensated cirrhosis and hepatocellular carcinoma [1,2,3,4]. Although a prophylactic vaccine is available to prevent infection, current antiviral therapies rarely achieve a cure for chronic HBV infection. Therefore, most HBV patients need to be on lifelong antiviral treatment. HBV has a very narrow host tropism, and only naturally and efficiently infects humans and chimpanzees. However, non-human HBV viruses are found in other organisms including mammals (orthohepadnaviruses), birds (avihepadnaviruses), reptiles and fish [5,6,7,8,9,10,11,12,13,14]. Two of these non-human HBV viruses that have impacted our understanding of the HBV life cycle the greatest are duck HBV (DHBV) and woodchuck HBV (WHBV) [15,16].

**Figure 1:**
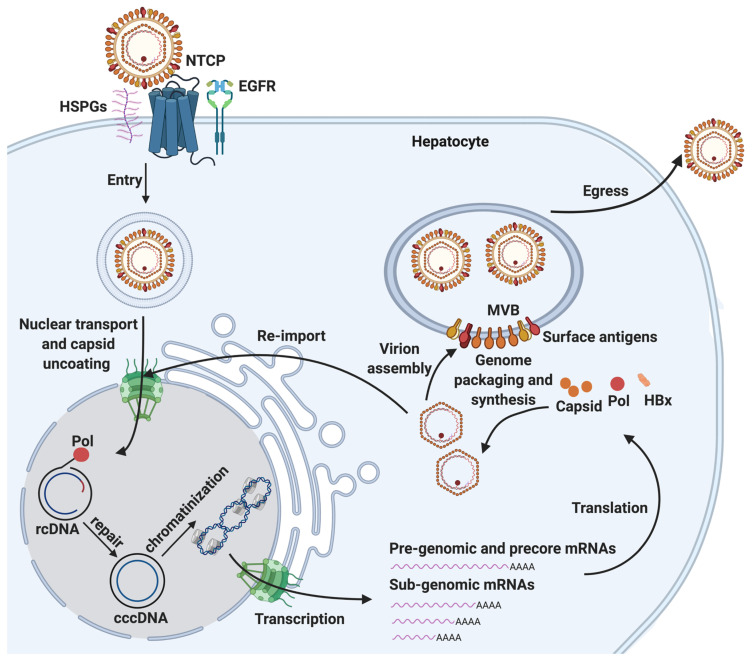
The HBV life cycle and cccDNA biogenesis. HBV life cycle is a multi-step process composed of viral entry, cccDNA biogenesis, progeny nucleocapsid production, virion formation, and egress. Viral entry is mediated by NTCP, heparin sulfate proteoglycans (HSPGs, such as glypican 5), and EGFR on the surface of hepatocytes. cccDNA biogenesis is an essential process to establish infection, which consists of three distinct steps: nuclear transport of rcDNA and uncoating, repair of rcDNA to form cccDNA, and cccDNA chromatinization. cccDNA can serve as the template for pre-genomic and precore mRNAs and multiple sub-genomic mRNAs, which can be translated to several viral proteins, including HBV POL, core antigen (capsid), surface antigens, HBx, and core-antigen related proteins (not shown). Progeny nucleocapsid production is initiated by the binding of HBV POL to pre-genomic mRNA, which triggers the packaging (encapsidation) and synthesis of rcDNA. The resultant nucleocapsids can either be re-imported into the nucleus, and the rcDNA repaired to form cccDNA and maintain cccDNA pool through the intracellular amplification pathway, or it can be enveloped in the multivesicular body (MVB) to complete virion assembly. Subsequent virion egress completes the HBV life cycle.

HBV is one of the smallest viruses and has a very compact 3.2 kb DNA genome with a very limited coding capacity. The genome is organized into four partially overlapping open reading frames (ORFs). These encode the four major gene products: (1) the viral polymerase POL, which is involved in viral replication and packaging [17,18,19]; (2) three HBV surface polypeptides (HBs), namely the small (S), medium (M), and large (L) surface antigens that are incorporated in the viral envelope and mediate viral entry [20]; (3) HBV core protein (HBc), which comprises the viral capsid (essential for viral replication and genome packaging) and several core-related proteins, including the secreted HBV e antigen (HBe) and a pre-core protein [20,21,22]; and (4) the X protein (HBx), which has been shown to have pleiotropic functions, such as the regulation of viral genome transcription [23,24,25,26]. Together with host factors, these viral proteins drive the completion of the HBV life cycle, which includes virus entry, biogenesis of cccDNA, progeny nucleocapsid production, virion formation and virion egress (Figure 1).

Viral entry is initiated via low-affinity binding of the virion to the cell surface heparin sulfate proteoglycans (HSPGs, such as such as glypican 5) [27,28], and the subsequent binding of the N-terminal region of the large HBs (PreS1) to the bile acid transporter sodium-taurocholate cotransporting polypeptide (NTCP), expressed on hepatocytes [29,30]. Following receptor-mediated endocytosis, the viral nucleocapsid containing a form of the HBV genome, rcDNA, is released into the cytoplasm of the host hepatocyte. Epidermal growth factor receptor (EGFR) has also been shown to facilitate this process [31,32,33].

**Figure 2:**
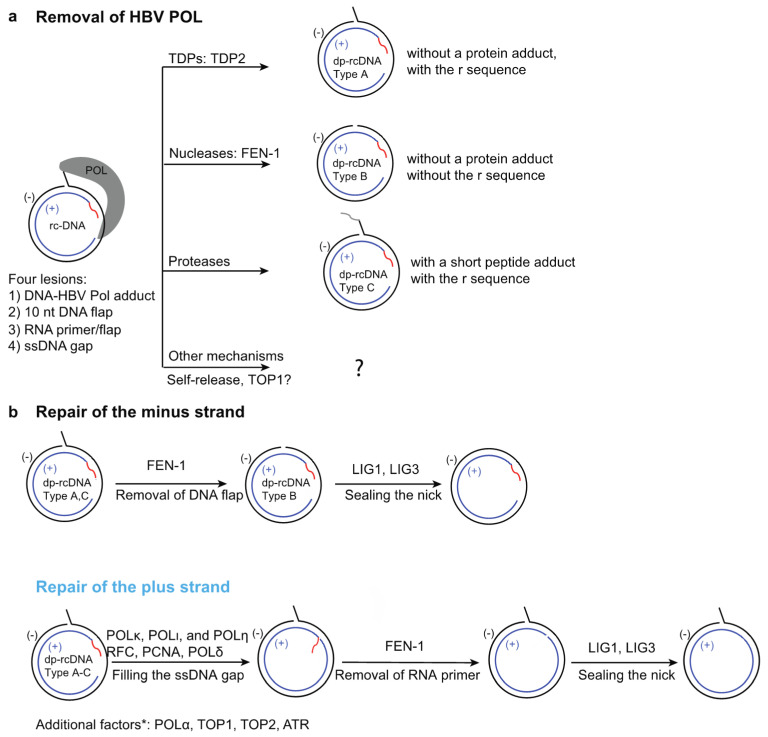
The repair factors and steps involved in conversion of HBV rcDNA to cccDNA. (**a**) Removal of HBV polymerase (POL) adduct from rcDNA can be achieved by: (1) tyrosylphosphodiesterases (TDPs), such as TDP2; (2) nucleases, such as FEN-1; (3) proteases; and (4) other mechanism such as self-release of POL, or TOP1 mediated release. The first three mechanisms will lead to formation of types A−C of deproteinated rcDNA (dp-rcDNA). Type A and C dp-rcDNAs contain the terminal redundancy DNA flap, whereas type B dp-rcDNA does not. The repair intermediates of POL self-release or TOP1-mediated release are not clear and are thus denoted by a question mark. (**b**) After the removal of POL, the minus strand is further repaired by removal of the terminal redundancy DNA flap via FEN-1 or other nucleases and ligation of the nick by LIG1 or LIG3. The steps involved in repair of the plus strand are: (1) completion of DNA synthesis by various host DNA polymerases; (2) removal of the displaced RNA primer via FEN-1; and (3) ligation of the nick by LIG1 and LIG3. Additional factors POLα, TOP1, and TOP2 are shown to be involved in cccDNA formation; however, it is not clear which steps they are involved in. ATR has been shown to be involved in preventing the degradation of the minus stand. Red wavy line, RNA primer; gray wavy line, remnant peptide post protease digestion.

HBV rcDNA within the capsid has a peculiar structure containing four distinct lesions: (1) the HBV polymerase POL covalently linked to the 5′ end of the minus strand through a tyrosylphosphodiester bond; (2) a terminal redundancy sequence (r) consisting of a ten nucleotide (nt) DNA flap on the minus strand; (3) a 5′-capped RNA primer; and (4) a single-stranded DNA (ssDNA) gap on the plus strand (Figure 2a). The incomplete nature of rcDNA renders it incompetent to serve as the template to produce all HBV viral mRNAs, and it needs to be converted into chromatinized cccDNA to establish infection.

cccDNA biogenesis is a complex multiple-step process, which involves the nuclear transport of rcDNA, rcDNA repair and cccDNA chromatinization (Figure 1). Each of these steps requires extensive and intricate interaction of viral components and host factors. The import of rcDNA from the cytoplasm into the nucleus likely involves a conformational change or the partial disassembly of capsid to display its NLS on the outside surface, which interacts with karyopherin α and β, and results in NPC localization of the nucleocapsid [34,35,36,37,38,39,40]. POL has also been shown to contain a bipartite NLS that could be exposed by casein kinase II (CKII)-dependent phosphorylation and interact with karyopherin α2, contributing to the import of rcDNA into the nucleus [41].

The repair of rcDNA to form cccDNA has been an enigma for decades, and elusive host repair machinery components play essential roles in this step. The resultant cccDNA is decorated with host nucleosomes and viral factors, such as HBc [42,43]. This cccDNA mini-chromosome is also subjected to a plethora of histone modifications [44] and serves as the transcriptional template for all viral mRNAs, including the pregenomic RNA (pgRNA), which is the template for the generation of the progeny HBV rcDNA. The interaction of HBV POL with a specific secondary structure (ε) on pgRNA triggers the formation of nucleocapsid, which matures through complex processes including reverse transcription, primer translocations and circularization steps inside the nucleocapsid [19,45,46,47]. The mature nucleocapsids can either be enveloped to form HBV virions, which are subsequently released from hepatocytes to complete the life cycle of HBV, or their rcDNA cargo can be re-imported into the nucleus to be converted into cccDNA to maintain a stable pool of cccDNA [15]. cccDNA biogenesis through re-import of progeny nucleocapsids is termed intracellular amplification, to distinguish from the aforementioned *de novo* biogenesis mode, through which cccDNA is formed via nucleocapsids from the incoming virions during infection. Differential repair factors have been implicated in these two modes of cccDNA biogenesis [33,48]; however, it is still unclear how else these two modes may differ from each other.

There is currently no cure for chronic HBV infection, and the targeted eradication of cccDNA is regarded as the holy grail of a cure [49,50]. One critical step toward this goal is to block cccDNA biogenesis, and the identification of host factors involved in rcDNA repair is essential to the discovery of potential therapeutic targets to combat HBV infection. During the past five to ten years, there have been numerous findings of host repair factors and molecular mechanisms involved in rcDNA repair. In this review, we will first briefly discuss the effects of viral proteins on cccDNA formation and then focus on the recent identification of host repair factors and repair mechanisms in rcDNA to cccDNA conversion. We will introduce the genetic and biochemical approaches used in these findings and then summarize the repair of each individual lesion of rcDNA, based on findings mainly about HBV and DHBV. We will also compare the differences of cccDNA biogenesis between these viruses. Lastly, we will summarize the effects and therapeutic potential of small molecule inhibitors targeting these repair factors on cccDNA formation. Since the remainder of this review will focus on rcDNA repair, we refer readers to previous reviews for additional details of the HBV life cycle and other steps of cccDNA biogenesis, including the nuclear transport of nucleocapsids and the chromatinization of cccDNA [15,45,51,52,53,54,55,56,57].

## 2. Functions of HBV Viral Factors in rcDNA Repair

HBV encoded proteins are critical for completion of the HBV life cycle and generate infectious HBV virions containing HBV rcDNA. rcDNA repair requires various repair enzymes, and the only viral protein which contains an enzymatic activity is POL [58,59,60]; however, the inhibition of the DNA polymerase activity of HBV POL does not affect HBV or DHBV cccDNA formation in cells [61,62,63,64,65]. Therefore, the viral proteins most likely play limited roles in rcDNA repair in cccDNA biogenesis. Consistent with this notion, the lack of surface antigens in HBV or DHBV does not lead to a decrease of cccDNA formation. On the contrary, the lack of surface antigen in DHBV results in a drastic increase of cccDNA levels, most likely due to the increased retention of rcDNA containing nucleocapsids, which increases the intracellular amplification of cccDNA [66,67,68,69]. HBc is an essential structural component of the nucleocapsid and is involved in two steps of cccDNA biogenesis: nucleocapsid transport and perhaps cccDNA chromatinization. It is not clear if HBc plays a direct role in the rcDNA repair step. HBV mutants deficient in producing core (HBV_∆c) or HBx (HBV_∆x) can establish cccDNA, indicating that *de novo* synthesis of HBV core or HBx is not required for *de novo* cccDNA biogenesis [62,70,71]. However, HBV_∆c virions generated via trans-complementation methods contain capsids and it is not clear if these preexisting HBc capsids play a direct role in the rcDNA repair step.

## 3. General Approaches and Challenges of Studying cccDNA Formation

The identification of repair factors and mechanisms by which rcDNA is repaired have been among the central questions in the HBV field for the past four decades. Addressing these questions in detail requires a combination of complementary approaches, including genetic analyses in cell culture systems and a biochemical reconstitution system. All experimental systems have their strengths and weaknesses, and a better understanding of the resolution, caveats and limitations of each approach are critical for the appropriate interpretation and generation of experimental lines of evidence.

There are several types of cell culture systems to study *de novo* cccDNA formation and intracellular amplification [72]. The identification of human NTCP (hNTCP) as one essential HBV entry factor led to the generation of various hNTCP-expressing human hepatoma cell lines that are susceptible to HBV infection [29]. Primary human or duck hepatocytes are permissive to HBV or DHBV infection, respectively, and these two types of cell culture systems are used in studying *de novo* cccDNA formation from incoming virions. On the other hand, HBV or DHBV hepatoma cell lines that stably produce pgRNA driven by a tetracycline-regulated promoter are routinely used to study cccDNA biogenesis via the intracellular amplification mode [73,74].

Since cccDNA formation is a dynamic process that relies on multiple cellular processes and host-HBV interactions, genetic studies utilizing the aforementioned cell lines are better suited to capturing the dynamics and factors involved. However, there are several challenges in genetic analyses using cell culture systems. Firstly, HBV infection and cccDNA formation in the hNTCP-expressing hepatoma cell lines is inefficient, requiring a very high multiplicity of infection (MOI) yet producing very few copies of cccDNA per cell. This low level of cccDNA formation has made it challenging to accurately determine cccDNA levels. Secondly, many DNA repair factors are essential in human cell lines, and thus bi-allelic inactivation hampers the generation of mutant cell lines. Thirdly, knockdown or knockout of DNA repair factors have pleiotropic effects on the cells, which may indirectly affect repair of rcDNA (e.g., altering cell cycle status) and may be erroneously identified as factors directly involved in cccDNA formation. Fourthly, some DNA repair factors are highly active, and their levels must be depleted to less than 10% in order to affect their functions [75]. These types of factors that are involved in cccDNA formation are very likely to be overlooked if near complete depletion is not achieved.

Biochemical approaches utilizing cell extracts or purified proteins have been used to study the repair of various types of lesions contained in rcDNA [50,76,77]. Recently, a biochemical system has been established that fully reconstitutes the repair of rcDNA substrates to form cccDNA [19,75]. Biochemical systems have four major advantages. Firstly, they confer easy and more accurate detection of cccDNA formation. Secondly, they can be used to examine of the function of essential repair factors. Thirdly, they can be used to directly examine the repair of each rcDNA repair step with purified substrates and repair factors. Fourthly, they allow the manipulation of concentrations of substrates and factors, as well as the sequential addition of factors, and are thus powerful tools for deciphering the detailed molecular mechanism of rcDNA repair. On the other hand, biochemical systems also have limitations. They cannot capture all of the complexity of the events that occur in cells, and since they require high purity of the substrates and protein factors, they are prone to contamination.

The strengths of these cell-based and biochemical approaches compensate for each other’s shortcomings. During the past ten years, the combination of these two approaches has produced exciting findings and has brought us closer to a more holistic picture of how rcDNA is repaired to form cccDNA.

## 4. General Steps Involved in rcDNA Repair

There are four lesions on both strands of rcDNA; therefore, it has been proposed that five individual repair steps need to occur to form cccDNA: (1) removal of HBV POL; (2) removal of the terminal redundancy sequence r; (3) complete cleavage of RNA primer; (4) completion DNA synthesis of plus strand; and (5) ligation of nicks on both strands (Figure 2). In the following sub-sections, we summarize the recent cell-based genetic and biochemical findings of repair factors and mechanisms involved in these five steps (Figure 2).

### 4.1. Removal of HBV POL from HBV rcDNA

HBV POL is covalently linked to the 5′ end of the minus strand via a tyrosylphosphodiester bond [78,79], and POL is engaged in partially extending the 3′ end of the plus-strand and likely prevents access of other factors to it [80,81]. Therefore, this protein adduct blocks the repair of both strands and needs to be removed first to generate de-proteinated rcDNA (dp-rcDNA) intermediates to initiate the repair process (Figure 2a). Various dp-rcDNA species without POL have been detected in various HBV cell culture models and infected liver samples [82,83,84,85,86,87,88,89]. It has been proposed that the deproteination step takes place in the cytoplasm and the dp-rcDNA are precursors for cccDNA formation; dp-rcDNA are transported into the nucleus, where the subsequent repair is completed and cccDNA is formed [83,88]. However, it is also conceivable that the long-lived dp-rcDNA detected in cells may be dead-end repair products and cannot be further processed to form cccDNA. The transfection of purified cytoplasmic DHBV dp-rcDNA into LMH chicken hepatoma cells led to the formation of DHBV replication intermediates, supporting the notion that DHBV dp-rcDNA intermediates can be converted into cccDNA [83]. However, similar experiments using HBV dp-rcDNA have not been reported, so it remains to be determined if HBV dp-rcDNA can be converted to cccDNA both in cells and biochemical systems. Theoretically, there are several ways to remove the HBV-POL adduct (Figure 2a): (1) tyrosyl-DNA phosphodiesterases (TDPs) that precisely remove the 5′-HBV POL adduct, resulting in the type A dp-rcDNA containing a 10 nt terminal redundancy flap with a 5′-P group; (2) removal of HBV POL and the terminal redundancy by a DNA endonuclease, generating the type B dp-rcDNA; (3) through protease digestion, generating the type C dp-rcDNA with a remnant peptide linked to the 5′ DNA end; and (4) other mechanisms including HBV POL self-release or topoisomerase-mediated release.

#### 4.1.1. POL Removal—Release by Tyrosyl-DNA Phosphodiesterases (TDPs)

TDPs are a family of enzymes that specifically act on and release the protein adducts that are covalently linked to DNA through either 3′-or 5′-tyrosylphosphodiester bonds [90]. In human cells, TDP1 and TDP2 can specifically cleave 3′-and 5′-tyrosylphosphodiester bonds respectively [76,91]. Since HBV POL is covalently linked to the 5′ end of the minus strand via a tyrosylphosphodiester bond, TDP2 seems to be a perfect candidate to remove HBV POL from rcDNA. Biochemically, purified TDP2 can remove DHBV and HBV polymerase from rcDNA isolated from nucleocapsids [76]. TDP2 can also remove the polymerase from recombinant substrates that contain DHBV or HBV polymerase covalently linked to a primer generated in in vitro protein priming reactions [76,92]. However, the effect of TDP2 on cccDNA biogenesis in cell culture systems is more complex to interpret. TDP2 knockdown or knockout leads to a 2–3 day delay of intracellular amplification of cccDNA in envelope deficient DHBV (DHBVenv−), as shown in HepG2 derivative cell lines [76,93]. Amplification and *de novo* HBV cccDNA formation were also reported to occur in TDP2 knockout cells and when TDP2 was suppressed with pharmacological inhibitors of the enzyme [93,94]; however, it is not clear whether cccDNA formation is also delayed, as reported for DHBV, since cccDNA formation time course experiments have not been reported to date [88,93]. Furthermore, the effects of human TDP2 overexpression on cccDNA levels are confounding. It has been shown that overexpression of shRNA-resistant TDP2 could partially rescue the delayed cccDNA amplification of DHBVenv− in TDP2 deficient HepG2 cells [76], but the opposite is observed for an equivalent envelope-deficient mutant HBVenv- in HEK293 cells [93]. While seemingly contradictory, these differences could be attributed to the fact that different cell lines were used and that repair of HBV and DHBV rcDNA may require different factors. Altogether, these findings indicate that TDP2 most likely plays a non-essential role in HBV or DHBV POL removal and that there are redundant factors or other mechanisms to remove HBV POL.

Additional findings about dp-rcDNA support the notion that TDP2-like enzymes are involved in POL removal. If the dp-rcDNA were authentic repair intermediates to form cccDNA, the DNA sequences at the termini of dp-rcDNA may provide insights into the removal mechanisms of POL. A recent study, which used the rapid amplification of cDNA ends (RACE) method, determined the terminal sequences of DNA ends in cytoplasmic dp-rcDNA in the HepDES19 cell line [88]. Cytoplasmic HBV dp-rcDNA contains type A dp-rcDNA (Figure 2a), which uniformly contains a phosphorylated 5′ end, indicating the complete removal of polymerase from rcDNA and requires enzymes with tyrosyl-phosphodiesterase activity, such as TDP2. This study also showed that TDP2 knockout did not affect the level of dp-rcDNA, suggesting that additional unknown TDPs play redundant roles. However, it has not been determined if the dp-rcDNAs in TDP2 knockout cells contain the terminal redundancy, and it is possible that other types of mechanisms instead of TDPs play redundant roles to TDP2 in POL removal. It is worth noting that the RACE method used in the study could not be used to detect type C dp-rcDNA (Figure 2a) with a remnant peptide adduct. The method may also not detect type B dp-rcDNA generated by endonucleases, as this type may be short-lived and is quickly ligated to the 3′ end of the minus strand, allowing it to evade detection. This scenario is supported by the fact that the FEN-1 mediated cleavage and LIG1 mediated ligation are highly coordinated, and it has been shown biochemically that the nick generated by FEN-1 is sealed by LIG1 in under a minute [19].

#### 4.1.2. POL Removal—Release by FEN-1 Endonuclease

FEN-1 is a structure-specific nuclease that processes substrates containing a 5′ flap and is critical in DNA replication and repair [95,96]. Several lines of biochemical and cell-based evidence have shown that FEN-1 plays a key role in POL removal. A recent study has established a biochemical system that supports HBV cccDNA formation in vitro using recombinant HBV rcDNA [75]. This recombinant HBV rcDNA is a close approximation to the authentic rcDNA and contains a NeutrAvidin-biotin-DNA adduct to mimic the HBV POL adduct. It is worth noting that it lacks a 5′-tyrosylphosphodiester covalent bond, and thus cannot be processed by TDP2. However, it is suitable for studying the alternative mechanisms of HBV POL removal. Using such a biochemical system, five factors that are involved in DNA lagging strand synthesis, namely proliferating cell nuclear antigen (PCNA), the replication factor C complex (RFC), DNA polymerase δ (POLδ), FEN-1, and DNA ligase 1 (LIG1), have been identified to be both sufficient and necessary to convert the recombinant rcDNA into cccDNA; as such, they comprise one minimal set of factors essential for rcDNA repair in vitro [75]. In the same study, it was shown that the flap endonuclease FEN-1 can remove the NeutrAvidin-biotin-DNA adduct from the recombinant rcDNA by cleaving the terminal redundancy flap DNA linked to the protein adduct. Interestingly, the activity of FEN-1 towards the flap DNA is greatly reduced by the presence of protein adducts compared to an adduct-free flap. This observation is consistent with previous findings that FEN-1 cleaves its flap substrates via a treading through the 5′ end of a flap structure and track mechanism, and thus a large protein adduct blocking the 5′ end of the flap substrate drastically slows down the reaction [97].

HBV cccDNA formation is inefficient in cell culture, which is possibly due to FEN-1′s low activity on rcDNA containing POL. A previous study has shown that, in hepatoma cells supporting HBV cccDNA amplification, ectopically expressed FEN-1 is mainly localized to the nucleus and interacts with HBV DNA [77]. Furthermore, a small molecule inhibitor of FEN-1, PTPD, can effectively inhibit *de novo* cccDNA formation and amplification [19,77,98,99]. These findings indicate that FEN-1 plays a critical role in the removal of HBV POL from rcDNA. It is worth noting that FEN-1 plays multiple roles in rcDNA repair. Other than the removal of HBV POL, FEN-1 also participates in removing the RNA primer on the plus strand and may function in removing the terminal redundancy from types A and C dp-rcDNA after TDP- and protease-mediated POL removal, see Section 4.2 and Section 4.3 for details (Figure 2).

#### 4.1.3. POL Removal—Release by Proteases

It has been shown that purified DHBV virions can generate a very small portion (~1%) of dp-rcDNA when subjected to an endogenous polymerase reaction (EPR) that further extends the incomplete plus strand [38]. The generation of the dp-rcDNA is dependent on near complete synthesis of the plus strand and an unknown serine protease, as the generation of dp-rcDNA is sensitive to treatments of the DHBV/HBV DNA polymerase inhibitor phosphonoformate and a serine protease inhibitor 4-(2-aminoethyl)benzenesulfonyl fluoride (AEBSF) [38]. However, it is unknown how the completion of the plus strand synthesis leads to proteolysis and the removal of DHBV POL. Two explanations have been proposed. The completion of plus strand synthesis leads to an increased negative charge of longer DNA, which can result in electrostatic repulsion. In addition, the completion of plus strand synthesis may also lead to changes of phosphorylation status of capsid and trigger its disassembly [40,100,101], thus granting access of rcDNA to a co-purified protease. Alternatively, the serine protease may be packaged in the virion and completion of plus strand synthesis triggers DHBV POL digestion through unknown mechanisms. However, it has not been tested whether a serine protease is also required in POL removal in purified HBV virions, and it is possible that POLs are removed differently in HBV and DHBV rcDNA.

#### 4.1.4. POL Removal—Additional Release Mechanisms

It has also been proposed that HBV POL can release itself from rcDNA. Since the covalent 5′ POL-DNA adduct is similar to the DNA-topoisomerase adduct, it seems possible that HBV POL can re-ligate these two strands and release itself in a fashion similar to that of topoisomerases [102]. However, due to the challenges in purifying HBV POL, this possibility has not been fully examined. TOP1 has also been implicated in removing POL by cleaving the minus strand of DHBV rcDNA [103], although further studies are required to validate these findings in HBV (see Section 4.2 for details).

### 4.2. Removal of the Terminal-Redundancy Sequence r

After the removal of POL, which blocks the repair of both strands, the repair of the minus and plus strands can proceed. The minus strand contains a 10 nt terminal-redundancy sequence r, which needs to be completely removed to prevent insertion and frame shifting mutations, which would lead to the formation of defective cccDNA molecules. Removal of the r sequence requires enzymes with nuclease activities and, since the sequence has a 5′ flap structure, it is an optimal substrate for FEN-1 endonuclease. Indeed, FEN-1 removes the 10 nt flap in recombinant types A&C dp-rcDNA substrate (Figure 2) within 1 min in biochemical assays, generating a nick that can be ligated to the 3′ end of minus strand by DNA ligase LIG1 [19]. It also appears that the removal of the flap and the subsequent ligation step are intricately coordinated, and the repair intermediate that lacks the flap is quickly ligated to the 3′ end of the minus strand, making it barely detectable in biochemical assays [19]. As mentioned in Section 4.1.2, FEN-1 can also remove a 5′ flap with a protein adduct to generate type B dp-rcDNA. In this case, the removal of POL and the terminal-redundancy sequence is executed by FEN-1 in a single reaction; however, this reaction is much less efficient compared to the removal of a protein free 5′ flap.

Human topoisomerase TOP1 has been shown to cleave the 3′ end of the minus strand of DHBV dp-rcDNA and circularize the minus strand with low efficiency in biochemical assays [103]. Recircularization requires a 5′-OH of the minus strand and is highly prone to deletion [103]. However, the HBV dp-rcDNA produced in human cell lines uniformly contains a 5′-P [88], and hTOP1-mediated cleavage of HBV dp-rcDNA has not been reported in biochemical assays. A recent cell culture-based study showed that TOP1 and TOP2 inhibitors reduced *de novo* cccDNA formation and its amplification [104]. A closer investigation revealed that inhibition of TOP1 or TOP2 diminished the repair of minus strand rcDNA. However, TOP1 knockdown experiments showed contradictory results, in which cccDNA formation was increased [104]. Therefore, future studies are required to clarify whether TOP1 plays a role in the removal of the r sequence and the recirculization of the minus strand of HBV rcDNA.

### 4.3. RNA Removal of HBV rcDNA

The plus strand of rcDNA resembles the structures of a DNA lagging strand during DNA synthesis. Two recent biochemical studies have shown that the repair of the plus strand resembles the process of the maturation of the Okazaki fragments, including RNA primer removal [19,75]. The ssDNA gap is equivalent to those between individual Okazaki fragments, while the 18 nt RNA primer is similar to those in Okazaki fragments. Only seven out of the 18 nt of the RNA primer on the plus strand anneal to the minus strand, generating a structure composed of 7 bp hetero-duplex and a 11 nt RNA flap. Similarly to a DNA flap, RNA flaps have also been shown to be substrates of FEN-1 [105]. FEN-1 alone can only partially remove this 18 nt RNA primer (most likely the 11 nt RNA flap) in a biochemical assay. This is presumably due to the remnant RNA primer forming a hetero-duplex with the minus strand, which is not a substrate for FEN-1. The full removal of the RNA primer requires displacement of the remnant RNA by complete synthesis of the plus strand, generating an RNA flap, which could be fully removed by FEN-1.

Theoretically, RNA primer could be removed by other factors containing nuclease activities similar to FEN-1 or enzymes containing RNase H activity. The latter includes HBV POL [106] and host factors such as RNaseH [107]. Future studies are required to identify the functions of these factors in RNA removal in rcDNA.

### 4.4. Completion of Synthesis of the Plus Strand

An in vitro study has shown that the complete synthesis of the plus strand and displacement of the RNA primer requires PCNA, RFC, POLδ [19]. As shown in (Figure 2b), the 3′ terminus of the plus strand is equivalent to a primer and can be recognized by the RFC complex, which loads the homotrimer clamp PCNA ring onto the rcDNA [108,109]. PCNA interacts with the POLδ complex through a PCNA-interacting peptide (PIP) sequence and serves as the processivity factor for POLδ complex [110,111]. PCNA-POLδ can complete the synthesis of the plus strand and displace the RNA primer to generate a flap to be removed by FEN-1, which generates a nick that could be sealed by DNA ligases. Consistent with this finding, aphidicolin, a small molecule inhibitor for DNA POLδ, α, and ε [112] reduces cccDNA formation in a biochemical system with POLδ as the only DNA polymerase, and in cell lines infected with HBV virus [19,75]. After a 24 h pre-treatment with 2% DMSO, treatments of 100–400 μM aphidicolin are well tolerated by cells for at least 60 h. Since *de novo* cccDNA formation from incoming viruses can be detected by Southern blot at 24–48 hrs after infection, cytotoxicity is not a complicating factor in these studies. A lower dose of aphidicolin (10 μM) can inhibit POLα, which is more sensitive to aphidicolin, but is not enough to inhibit POLδ activity [75,113]. Therefore, it is not surprising that 10 μM aphidicolin does not reduce cccDNA formation in biochemical assays or cells infected with HBV [62,75]. POLδ has also been shown to be involved in cccDNA formation through intracellular amplification [33]. A previous study generated a POLδ hypomorphic cell line by CRISPR, in which one allele has a frameshift mutation and the other has a 4 aa in-frame deletion in a region that does not harbor catalytic residues [33]. This POLδ hypomorphic cell line was shown to have a reduced level of cccDNA amplification. Interestingly, the same study also found that POLα plays a bigger role than POLδ in cccDNA amplification and proposed that POLα participates in a step of the minus strand repair before ligation, instead of the elongation of the plus strand. Further studies are required to elucidate the detailed function of POLα in cccDNA formation.

Multiple other host DNA polymerases have been implicated in the completion of DNA synthesis of the plus strand, including translesion DNA polymerases POLι, POLη, and POLκ, with the latter playing the most important role among these three factors [62]. POLκ knockdown or knockout in HBV-susceptible, hNTCP-expressing HepG2 cell lines results in reduced cccDNA levels following HBV infection. However, two independent studies showed that siRNA knockdown of POLκ does not affect cccDNA formation through the intracellular amplification route [33,50]. It is not clear if this difference between *de novo* cccDNA formation and its amplification is due to the factors and mechanisms being different between these two pathways, and future studies are required to clarify this. It is not unusual that multiple polymerases participate in various DNA replication and repair pathways. For example, POLα, POLδ, and POLε are all required in eukaryotic DNA replication [114,115], and previous studies have shown that nucleotide excision repair in human fibroblasts requires POLδ, POLκ, and POLε [116]. Therefore, it is conceivable that POLδ, POLκ, POLι, and POLη play redundant roles in completion of HBV plus strand synthesis.

It is worth noting that the POL of various HBV-like viruses has been shown to have DNA polymerase activity and can extend the incomplete plus strand of rcDNA in the capsids [58,59,60]. However, inhibition of the DNA polymerase activity of viral POL does not block the cccDNA formation of HBV and DHBV, indicating that POL is dispensable in rcDNA repair [61,62,63,64,65]. These findings indicate that the completion of DNA synthesis of the plus strand repair probably results from a concerted effort of both host and viral polymerases.

### 4.5. Ligation of Nicks on Both Strands

There are three DNA ligases in human cells: LIG1, LIG3, and LIG4 [117,118,119,120,121]. LIG1 is involved in the ligation steps of multiple processes, including Okazaki fragment maturation during DNA lagging strand synthesis, homologous recombination repair (HR), long-patch base-excision repair (BER) and nucleotide excision repair (NER) [117]. LIG3 has various spliced forms, and is found in mitochondria and nucleus [117]. LIG3 is involved in ligating single strand DNA breaks and has been reported to share redundant roles with LIG1 [117,118,119,120,121]. LIG4 is a key component in non-homologous end joining (NHEJ) [117]. Purified recombinant human LIG1 has been shown to ligate the nicks on both the minus and plus strands and support cccDNA formation in biochemical reactions [19]. Cell based genetic analyses have shown that shRNA-mediated single knockdown of LIG1 and LIG3 diminishes both *de novo* cccDNA formation and its amplification; the effect on the latter is also confirmed in single knockout cell lines of LIG1 and LIG3 [50]. On the other hand, LIG4 is specifically involved in converting the double-stranded linear DNA molecules that are a common byproduct of rcDNA formation from pgRNA into a cccDNA-like molecule through the NHEJ pathway [50,122]. Since this pathway rarely processes the terminal redundancy correctly and is error prone, the resultant cccDNA-like molecules are likely defective. Although it has not yet been directly shown that LIG3 can covert rcDNA substrate to cccDNA in biochemical assays, LIG1 and LIG3 likely play redundant roles in sealing the nicks in both strands of rcDNA.

## 5. DNA Damage Response and HBV rcDNA Repair

The DNA damage response (DDR) detects various DNA lesions and coordinates extensive cellular programs to promote recovery after damage and to maintain genome integrity [123,124]. DDR relies heavily on various forms of posttranslational modifications (PTMs) that can quickly and reversibly change many protein properties and affect multiple cellular processes at once. The best understood PTMs in DDR are phosphorylation cascades mediated by the apical checkpoint kinases; the ataxia telangiectasia mutated (ATM) and Rad3-related (ATR) [125,126]. ATM and ATR are triggered by different types of DNA lesions. ATM is primarily activated by DNA double stranded breaks (DSBs), whereas ATR activation is mainly triggered by ssDNA binding protein complex RPA-coated ssDNA. The activation of ATM and ATR lead to the phosphorylation and activation of numerous substrates, including effector kinases CHK2 and CHK1, respectively. Such substrates mediate a cascade of downstream phosphorylation events to elicit a multitude of cellular responses including cell–cycle arrest, DNA repair, and apoptosis.

Many viruses have extensive interactions with DDR, and the life cycles of many DNA viruses have been shown to engage host DDR components [127,128,129,130], including HBV [72,131,132]. Recently, it has been shown that HBV infection and replication can activate the ATR-CHK1 branches of DDR, most likely through the DNA lesions on rcDNA [133]. In this study, inhibitors of ATR and its effector kinase CHK1, but not the ATM branch of DDR, significantly dampen the *de novo* formation and amplification of cccDNA in cells. Interestingly, ATR and CHK1 inhibitors lead to extensive degradation of the 5′ terminus of the minus strand of HBV rcDNA. These findings indicate that the ATR-CHK1 branch of DDR plays an important role in cccDNA formation, either by promoting the repair process or protecting the repair intermediates from degradation. However, it is not clear what downstream substrates are modulated by ATR/CHK1 activation to facilitate rcDNA repair or how DNA checkpoint response affects each individual repair step. Future studies are needed to answer these questions.

## 6. Differences in cccDNA Formation of HBV and DHBV

The biogenesis of cccDNA is most extensively studied in HBV and DHBV. There are several differences among the cccDNA levels and the regulation of cccDNA biogenesis of HBV and DHBV. It has been shown that the levels of DHBV cccDNA are much higher than those of HBV [68,76]. Single-cell analysis of duck hepatocytes chronically infected with DHBV indicated 1–36 copies of cccDNA/cell, with a mean value of ten copies/cell, and a broad range of distribution among individual cells and fluctuation during different stages of infection [134]. Copy numbers of cccDNA in liver biopsies of human chronic hepatitis B patients are reported to be 0.01–10 per cell, with a very large sample-to-sample variation [135,136]. Moreover, it has been shown that the DHBV cccDNA level in human hepatoma cell lines is still much higher than that of HBV cccDNA, indicating that virus-specific factors may influence the level of cccDNA [68,76]. It has been shown that the repair of an rcDNA-like molecule, which contains all the structures of DNA lesions but no HBV DNA sequences, can still be repaired to form a cccDNA-like molecule in biochemical assays, suggesting that the repair factors recognize the structures of lesions but not the sequence of rcDNA [75]. In light of these findings, the level of cccDNA formation between HBV and DHBV is most likely due to certain viral proteins and not viral sequences. Indeed, it has been shown that the envelope protein plays a critical role in regulating DHBV cccDNA level by preventing nuclear re-import of the nucleocapsid, and blocking the envelope protein production can lead to drastic increases of DHBV cccDNA level [66,67,68,69]. However, the HBV envelope proteins play a much less prominent role in controlling the HBV cccDNA level [68,69]. In addition, capsid disassembly is a critical step of cccDNA biogenesis. It has been shown that the capsid of HBV is more stable than that of DHBV [76], and it is likely that the viral core protein also contributes to differences in cccDNA formation between HBV and DHBV.

## 7. cccDNA Biogenesis in Murine Cells

HBV exhibits strict host tropism and only efficiently infects humans and chimpanzees. It is desirable to have small animal models to study HBV related pathogenesis, immune response and tumorigenesis. Although considerable advances have been made in various HBV animal models [8], the field still lacks an immune-competent HBV-susceptible mouse model. Two major blocks to establish HBV infection in mouse cells are viral entry and cccDNA biogenesis. Human NTCP (hNTCP) has been shown to be a critical entry factor for HBV and its satellite virus HDV in human hepatoma cells, and ectopic expression of hNTCP in human hepatoma cell lines results in HBV entry, cccDNA formation, and viral replication [29]. However, murine NTCP (mNTCP) does not permit entry of HBV or HDV [137]. Mutating mNTCP residues 84–87 to their human counterparts in murine hepatoma cell lines or live mice permits HBV and HDV entry; however, HBV cccDNA does not form and no productive HBV replication is detected [137,138]. Therefore, cccDNA biogenesis in murine cells is likely the final barrier in the generation of an immune-competent HBV-susceptible mouse model. A recent study showed that heterokaryonic murine and human hepatoma cells were permissive to HBV infection [139]. Although cccDNA formation was not directly examined by Southern blot in this study, the expression of HBeAg was used as a surrogate marker for cccDNA formation. Therefore, murine cells most likely lack certain factors to support cccDNA biogenesis. cccDNA biogenesis is a multi-step process and future cell-based genetic and biochemical studies are required to clarify whether any factors are missing in murine cells to support rcDNA repair or other steps in cccDNA biogenesis after HBV infection. It is worth noting that cccDNA can be detected in Hepatocyte Nuclear Factor 1 (HNF1)-null HBV transgenic mice, in which an over-length HBV genome is integrated into the chromosome [140]. In addition, cccDNA can be detected in an AML12 murine hepatic cell line derived from a transforming growth factor-alpha (TGF-α) transgenic mouse containing a Tet-inducible HBV integrate [141,142]. It remains to be determined how cccDNA can form in these murine cells and if HNF1-null mice or AML12 cell line expressing hNTCP can support *de novo* cccDNA formation upon HBV infection. Answering these questions will facilitate the development of immune-competent HBV-susceptible mouse models in the future.

## 8. Targeting DNA Repair Machinery as a Potential Treatment for HBV Infection

The inhibition of cccDNA formation and the eradication of the cccDNA pool is essential for a cure for HBV infection [49]. Understanding the factors and mechanisms involved in cccDNA formation can reveal novel targets for inhibiting cccDNA biogenesis. So far, it has been shown that cccDNA can be reduced by a handful of small molecule inhibitors that target various repair factors (Table 1). These include: aphidicolin, which inhibits the B family DNA polymerases [19,75,104]; a peptide derived from the cyclin-dependent kinase inhibitor p21, which disrupts PCNA and POLδ interaction [19,110,143]; FEN-1 endonuclease inhibitor PTPD [19,77]; topoisomerase inhibitors [104]; DNA ligase inhibitors [50]; and inhibitors against DNA checkpoint kinase ATR and CHK1 [133]. As many of these factors are essential for proliferating cells, it is challenging to target the host DNA repair pathway. However, since human hepatocytes are mostly quiescent cells [144], inhibiting certain DNA repair factors in hepatocytes may have minimal cytotoxic effects. Several strategies have been proposed to target hepatocytes and minimize side effects [145,146]. These strategies include hepatocyte-targeted delivery and the activation of pro-drugs via liver resident enzymes. It is likely that a combination of these approaches will lead to the generation of potent therapeutics that inhibit cccDNA levels by targeting host repair factors and the DDR pathway.

## 9. Concluding Remarks

Although HBV was discovered more than 50 years ago, several fundamental aspects of the viral life-cycle remain incompletely understood. These knowledge gaps have hindered progress towards a cure for chronic HBV infection. Although tireless efforts by many groups in the HBV field have contributed to a better understanding of the mechanisms underlying cccDNA formation, and have shown it to be a key step for HBV to establish persistent viral infection, numerous questions remain unanswered and other questions have arisen. For example, what is the complete set of host factors necessary for cccDNA formation in cells? Do some of these factors have redundant roles? Do auxiliary factors exist that possibly modulate the rate of rc- to cccDNA conversion? Do polymorphisms exist in any of the known or unknown factors, such as the DNA polymerases, RFC, FEN1, TDP2, or LIG1, and do they confer resistance to HBV within the human population? Do orthologues of these and other host factors from other mammalian species function sufficiently well to catalyze the rc- to cccDNA conversion reaction in non-human cells? How is the cccDNA pool maintained and what are the relative contributions of cccDNA stability and nuclear re-import of nascent rcDNA? To answer these and related questions, existing tools and methods need to be refined and new experimental approaches fearlessly pursued.

Before translating any of the findings we discussed in this review into potential novel therapies, it would be important to determine whether interference with, or complete abrogation of, cccDNA formation would have measurable effects on an already established HBV infection. If we are striving for an HBV cure, should we take the traditional risk-averse approach which often prevails when selecting targets for novel antiviral therapy, or might (transient) inhibition of host factors that are essential for cellular processes hold the key to a cure for chronic hepatitis B?

## Figures and Tables

**Table 1:** Reported inhibitors of various DNA repair factors that reduce cccDNA levels in biochemical and cell culture assays.

Inhibitor	Target	Effects on cccDNA Biogenesis	Effective Dose Tested	System Used	References
Aphidicolin	DNA polymerases POLδ, POLα, and POLε	Specifically inhibits the synthesis of the plus strand	100 μM	Biochemical	[19,75]
		Reduced *de novo* cccDNA formation and amplification	100–400 μM for *de novo* formation; 1 μM for intracellular amplification	hNTCP-HepG2 and HepAD38 cell lines	[33,75]
p21 peptide	PCNA-POLδ interaction	Specifically inhibits the synthesis of the plus strand	100 μM	Biochemical	[19]
PTPD	FEN-1 endonuclease	Reduced *de novo* cccDNA formation and its amplification	5–20 μM	hNTCP-HepG2 and Hep38.7-Tet cell lines	[19,77]
Topotecan	TOP1	Reduced cccDNA intracellular amplification	0.1–4 μM	HepAD38	[104]
Camptothecin	TOP1	Same as above	0.06–2 μM	HepAD38	[104]
Idarubicin	TOP2	Same as above	16–250 nM	HepAD38	[104]
Doxorubincin	TOP2	Same as above	62–250 nM	HepAD38	[104]
Aclarubicin	TOP2	Same as above	250–1000 nM	HepAD38	[104]
Mitoxantrone	TOP2	Same as above	500 nM	HepAD38	[104]
Merbarone	TOP2	Same as above	6–100 μM	HepAD38	[104]
L1	LIG1 and LIG3	Inhibits cccDNA formation	20 μM	Biochemical	[50,147,148]
L25	LIG1 and LIG3	Inhibits cccDNA formation	25 μM	Biochemical	[50,147,148]
L189	LIG1, LIG3, and LIG4	Inhibits cccDNA formation	50 μM	Biochemical	[50,147,148]
	LIG1, LIG3, and LIG4	Reduced cccDNA amplification in cell culture	10–20 μM	Tet- inducible HepDG10 cells	[50,147,148]
AZD6738	ATR	Reduced *de novo* cccDNA formation and intracellular amplification	25–50 μM	hNTCP-HepG2, AML12HBV10, and primary human hepatocytes	[133]
VE-821	ATR	Reduced *de novo* cccDNA formation and intracellular amplification	5–10 μM	hNTCP-HepG2, AML12HBV10	[133]
CGK733	ATM and ATR	Reduced *de novo* cccDNA formation	1–12 μM	hNTCP-HepG2, and primary human hepatocytes	[133]
Torin2	ATM and ATR	Reduced *de novo* cccDNA formation and intracellular amplification	0.03–1 μM	hNTCP-HepG2, AML12HBV10, and primary human hepatocytes	[133]
PF477736	CHK1 and CHK2	Reduced cccDNA intracellular amplification	8 μM	AML12HBV10	[133]
CHIR-124	CHK1	Reduced *de novo* cccDNA formation and intracellular amplification	1–4 μM	hNTCP-HepG2, HepAD38, AML12HBV10, and primary human hepatocytes	[133]

## Data Availability

Not applicable.
